# Supplementary material for: Topologically-based parameter inference for agent-based model selection from spatiotemporal cellular data
Source: PLoS Comput Biol. 2026 Sep 25;22(9):e1014801. doi: 10.1371/journal.pcbi.1014801 (PMC13641708; doi:10.1371/journal.pcbi.1014801)
Supplement: Supplementary file 1 [file pcbi.1014801.s001.pdf]

S1 Appendix. D'Orsogna and Alignment models

Supporting Information for "Topologically-based parameter inference for agent-based model selection from spatiotemporal cellular data."

Alyssa R. Wenzel, Patrick M. Haughey, Kyle C. Nguyen, John T. Nardini, Jason M. Haugh, and Kevin B. Flores

D'Orsogna model

The D'Orsogna model originally published in [1] is a mathematical framework used to describe the dynamics of self-propelled particles, such as those found in biological systems like swarming or flocking behaviors. It involves parameters that govern attraction and repulsion among agents. The system of ordinary differential equations modeling the D'Orsogna swarm behavior for $1 \leq i, j \leq N$ agents are detailed in (1).

$$\begin{aligned} \dot{\mathbf{x}}_i &= \mathbf{v} \\ m\dot{\mathbf{v}}_i &= \left(\alpha - \beta\|\mathbf{v}_i\|^2\right)\mathbf{v}_i - \nabla_{\mathbf{x}_i} U_i \\ U_i &= \sum_{j\neq i}^{N} C_r e^{-\|\mathbf{x}_j-\mathbf{x}_i\|/l_r} - C_a e^{-\|\mathbf{x}_j-\mathbf{x}_i\|/l_a} \end{aligned} \tag{1}$$

In the D'Orsogna model, α and β represent the self-propulsion and friction for each agent, respectively. We have fixed α=1 and $\beta = 0.5$ to be consistent with past work [1–3]. If no other forces are acting on an agent, the agent will asymptotically approach a constant speed of $\|\mathbf{v}_i\| = \sqrt{\alpha/\beta}$. In capturing the repulsion and attraction experienced by each cell, we use a Morse potential U_i for the ith agent. The Morse potential, while originally used to describe the potential energy of a diatomic molecule, provides a unique advantage in that it details the energy of an anharmonic oscillator; i.e., agents very close to $\mathbf{x}_i$ will produce a strong repulsive effect, but far away cells will produce a weakening attractive effect. The parameters representing the repulsive and attractive magnitudes are C_r and C_a, respectively, and the parameters accounting for the spatial sensitivity of repulsion and attraction are l_r and l_a, respectively. Often the ratios $\mathrm{C} \coloneqq C_r/C_a$ and $\mathrm{L} \coloneqq l_r/l_a$ are used in the plots of this paper and the phase diagram of Morse potential in D'Orsogna's paper. In a system of two particles, the zero force steady state distance is

$$\|\mathbf{x}_j - \mathbf{x}_i\| = l_r \frac{\ln(\mathrm{C}/\mathrm{L})}{1-\mathrm{L}} = l_a \mathrm{L} \frac{\ln(\mathrm{C}/\mathrm{L})}{1-\mathrm{L}}.$$

For there to be a physically meaningful steady state distance between two particles, we require that $\mathrm{L} < 1 < \mathrm{C}$ or $\mathrm{C} < 1 < \mathrm{L}$. For $\mathrm{C} < 1 < \mathrm{L}$, the Morse potential exhibits a maximum and there is no stable equilibrium. D'Orsogna et al. further describe in [1] systems beyond the parameters of (C, L) that are *H-stable* and *catastrophic*. The long-term patterns of H-stable systems scale with the number of particles; catastrophic systems collapse as the number of particles increase to infinity. Furthermore, the H-stable regions for their model must satisfy the following conditions: (I) $\mathrm{C} > 1$ and (II) lie above the separatrix $\mathrm{CL}^2 = 1$; all other regions are catastrophic.

Alignment model

In (1), we define the force of motion for an agent as

$$\mathbf{F}_i \coloneqq (\alpha - \beta\|\mathbf{v}_i\|^2)\mathbf{v}_i - \nabla_{x_i} U_i. \tag{2}$$

For our implementation, we seek to retain the primary forces which act on an agent; however, we want to extend the D'Orsogna model to include *alignment* $\mathbf{A}_i$ as an additional factor which changes velocity. We model alignment to fulfill the following assumptions:

- **Fluidization**: Neighboring agents will seek to move in parallel even if they are traveling in opposite directions. Perpendicular motion is the only instance where $\mathbf{A}_i = 0$.
- **Momentum**: Agents are given their own mass, m_i. Agents with greater momentum are more difficult to align; neighboring agents with greater momentum are more efficacious in affecting alignment.
- **Collective**: An agent's alignment is more greatly affected by the total alignment of all neighboring agents within a radius R.

We define the sensitivity to alignment as $\mathrm{W} \geq 0$. We define $0 \leq \Delta\theta_{in} \leq \pi$ as the angle between the agent vector $\mathbf{v}_i$ and its neighbor $\mathbf{v}_n$. Let $\chi_{2R}(\|\mathbf{x}_n - \mathbf{x}_i\|)$ be the characteristic function of a neighboring agent $\mathbf{x}_n$ being within a radial distance R from the agent $\mathbf{x}_i$. Therefore, we can model our assumptions in the following way

$$\begin{aligned} \mathbf{A}_i &= \mathrm{W}\sum_{n=1}^{N} \chi_{2R}(\|\mathbf{x}_n - \mathbf{x}_i\|)\cos(\Delta\theta_{in})\mathbf{v}_n \\ &= \mathrm{W}\sum_{n=1}^{N} \chi_{2R}(\|\mathbf{x}_n - \mathbf{x}_i\|)\frac{\|\mathbf{v}_n\|}{\|\mathbf{v}_i\|}\mathrm{proj}_{\mathbf{v}_n}\mathbf{v}_i \end{aligned} \tag{3}$$

Accounting for the mass of each agent m_i, we include the 2D components of alignment and set up a polar representation of acceleration.

$$\begin{aligned} \begin{pmatrix} \dot{v}_{x,i} \\ \dot{v}_{y,i} \end{pmatrix} &= \frac{1}{m_i}\begin{pmatrix} F_{x,i} \\ F_{y,i} \end{pmatrix} + \begin{pmatrix} A_{x,i} \\ A_{y,i} \end{pmatrix} \\ &= \begin{pmatrix} \cos(\theta_i) & -\sin(\theta_i) \\ \sin(\theta_i) & \cos(\theta_i) \end{pmatrix}\begin{pmatrix} \dot{r}_i \\ r_i\dot{\theta}_i \end{pmatrix} \end{aligned} \tag{4}$$

When solving for $\dot{r}_i$ and $r_i\dot{\theta}_i$, it becomes clear that in the current formulation, alignment provides some change in the magnitude of velocity. Therefore, if we want to retain the assumption that the D'Orsogna forces are the only forces that change the magnitude of velocity of an agent, we need to scale alignment such that $\dot{r}_i = 0$. In order to project the alignment $\mathbf{A}_i$ onto the $r_i\dot{\theta}_i$-space, our scaled alignment contribution is now

$$\begin{aligned} \dot{\mathbf{v}}_i &= \frac{1}{m_i}\mathbf{F}_i + \mathbf{S}(\theta_i)\mathbf{A}_i \\ \mathbf{S}(\theta_i) &= \begin{pmatrix} \sin^2(\theta_i) & -\cos(\theta_i)\sin(\theta_i) \\ -\cos(\theta_i)\sin(\theta_i) & \cos^2(\theta_i) \end{pmatrix} \end{aligned} \tag{5}$$

References

[1] Maria R D'Orsogna, Yao-Li Chuang, Andrea L Bertozzi, and Lincoln S Chayes. Self-propelled particles with soft-core interactions: patterns, stability, and collapse. *Physical review letters*, 96(10):104302, 2006.

[2] Kyle C. Nguyen, Carter D. Jameson, Scott A. Baldwin, John T. Nardini, Ralph C. Smith, Jason M. Haugh, and Kevin B. Flores. Quantifying collective motion patterns in mesenchymal cell populations using topological data analysis and agent-based modeling. *Mathematical Biosciences*, 370:109158, 2024. ISSN 0025-5564.

[3] Dhananjay Bhaskar, Angelika Manhart, Jesse Milzman, John T. Nardini, Kathleen M. Storey, Chad M. Topaz, and Lori Ziegelmeier. Analyzing collective motion with machine learning and topology. *Chaos: An Interdisciplinary Journal of Nonlinear Science*, 29(12):123125, 12 2019. ISSN 1054-1500. doi: 10.1063/1.5125493. URL https://doi.org/10.1063/1.5125493.
