# Supplementary material for: Topologically-based parameter inference for agent-based model selection from spatiotemporal cellular data
Source: PLoS Comput Biol. 2026 Sep 25;22(9):e1014801. doi: 10.1371/journal.pcbi.1014801 (PMC13641708; doi:10.1371/journal.pcbi.1014801)
Supplement: Supplementary file 2 [file pcbi.1014801.s002.pdf]

S2 Appendix. Experimental set-up

Supporting Information for "Topologically-based parameter inference for agent-based model selection from spatiotemporal cellular data."

Alyssa R. Wenzel, Patrick M. Haughey, Kyle C. Nguyen, John T. Nardini, Jason M. Haugh, and Kevin B. Flores

For each model simulation, we used the same fixed initial condition for position and velocity for each cell. This was randomly generated from a uniform distribution. Each simulation had the same length of time ($t_0 = 1, t_f = 21, \Delta t = 1/6$) and the same number of agents ($\#_{agents} = 300$). When calculating the Crocker plots, we used the last 100 time points of the simulations. The total time was 120 time points and the first 10 time points were used as a burn-in and the second 10 time points were used to calculate the velocity of each cell.
