# Supplementary material for: Topologically-based parameter inference for agent-based model selection from spatiotemporal cellular data
Source: PLoS Comput Biol. 2026 Sep 25;22(9):e1014801. doi: 10.1371/journal.pcbi.1014801 (PMC13641708; doi:10.1371/journal.pcbi.1014801)
Supplement: Supplementary file 3 [file pcbi.1014801.s003.pdf]

S3 Appendix. Statistical evaluation for model comparison

Supporting Information for "Topologically-based parameter inference for agent-based model selection from spatiotemporal cellular data."

Alyssa R. Wenzel, Patrick M. Haughey, Kyle C. Nguyen, John T. Nardini, Jason M. Haugh, and Kevin B. Flores

Permutational Multivariate Analysis of Variance

Permutational Multivariate Analysis of Variance (PERMANOVA) is a nonparametric statistical test used to assess differences between groups based on multivariate data. Unlike traditional analysis of variance (ANOVA), which relies on assumptions of multivariate normality and homogeneity of variances, PERMANOVA operates directly on a distance or dissimilarity matrix, making it well suited for complex, high-dimensional, or non-Euclidean data.

PERMANOVA partitions the total variation in the distance matrix into within-group and between-group components and computes a pseudo-F statistic analogous to the classical ANOVA F-statistic. Given a distance matrix D, the pseudo-F statistic is defined as

$$F = \frac{SS_{\text{between}}/(g-1)}{SS_{\text{within}}/(n-g)},$$

where SS_{between} and SS_{within} are the sums of squares associated with between-group and within-group variation, respectively, g is the number of groups, and n is the total number of observations.

Statistical significance is assessed through a permutation procedure rather than reliance on a known parametric distribution. Group labels are randomly permuted a large number of times, and the pseudo-F statistic is recomputed for each permutation. The resulting empirical distribution is used to estimate a p-value as

$$p = \frac{1 + \sum_{i=1}^{N} \mathbb{I}(F_i \geq F_{\text{obs}})}{1+N},$$

where F_{obs} is the observed pseudo-F statistic, F_i denotes the statistic from the i-th permutation, N is the total number of permutations, and $\mathbb{I}(\cdot)$ is the indicator function. For our test, we used N=999 permutations.

Because PERMANOVA is based on distances, its results depend on the choice of distance metric, and it is sensitive to differences in group dispersion. Nonetheless, it provides a flexible and robust framework for testing group-level differences in multivariate settings and is commonly used in ecological, biological, and other data-intensive applications.

Energy distance statistical test

The Energy Distance test is a nonparametric statistical test used to assess whether two samples are drawn from the same underlying probability distribution. It is based on the

concept of statistical energy and provides a measure of dissimilarity between distributions that is sensitive to differences in location, scale, and overall distributional shape. The test is particularly well suited for multivariate data and does not rely on parametric assumptions such as normality.

Let $X = \{X_1, \ldots, X_n\}$ and $Y = \{Y_1, \ldots, Y_m\}$ denote two independent samples in $\mathbb{R}^d$. The population energy distance between the distributions of X and Y is defined as

$$\mathcal{E}(X, Y) = 2\,\mathbb{E}\|X - Y\| - \mathbb{E}\|X - X'\| - \mathbb{E}\|Y - Y'\|,$$

where X' and Y' are independent and identically distributed copies of X and Y, respectively, and $\|\cdot\|$ denotes the Euclidean norm.

An unbiased sample-based estimator of the energy distance is given by

$$\hat{\mathcal{E}}_{n,m} = \frac{2}{nm}\sum_{i=1}^{n}\sum_{j=1}^{m}\|X_i - Y_j\| - \frac{1}{n(n-1)}\sum_{i\neq i'}\|X_i - X_{i'}\| - \frac{1}{m(m-1)}\sum_{j\neq j'}\|Y_j - Y_{j'}\|.$$

Under the null hypothesis that the two samples are drawn from the same distribution, the energy distance is zero. Statistical significance is assessed using a permutation procedure in which sample labels are randomly reassigned and the test statistic is recomputed for each permutation. The resulting empirical distribution is used to estimate a p-value in an analogous manner to other permutation-based tests.

The Energy Distance test is consistent against all fixed alternatives and is applicable in arbitrary dimensions, making it a powerful tool for detecting distributional differences in high-dimensional settings. Its nonparametric nature and reliance on pairwise distances align well with simulation-based and likelihood-free inference frameworks.

Maximum mean discrepancy test

The Maximum Mean Discrepancy (MMD) is a nonparametric statistical test used to determine whether two samples are drawn from the same underlying probability distribution. It is a kernel-based measure of discrepancy between distributions and is particularly effective for detecting differences in complex, high-dimensional data. The MMD test does not rely on parametric assumptions and is well suited for simulation-based and likelihood-free inference settings.

Let $X = \{X_1, \ldots, X_n\}$ and $Y = \{Y_1, \ldots, Y_m\}$ denote two independent samples drawn from distributions P and Q, respectively. Given a reproducing kernel Hilbert space $\mathcal{H}$ with associated positive-definite kernel $k(\cdot, \cdot)$, the population MMD is defined as

$$\mathrm{MMD}(P, Q) = \left\|\mathbb{E}_{X\sim P}[k(X, \cdot)] - \mathbb{E}_{Y\sim Q}[k(Y, \cdot)]\right\|_{\mathcal{H}}.$$

An equivalent squared formulation that is more convenient for computation is given by

$$\mathrm{MMD}^2(P, Q) = \mathbb{E}_{X,X'}[k(X, X')] + \mathbb{E}_{Y,Y'}[k(Y, Y')] - 2\,\mathbb{E}_{X,Y}[k(X, Y)],$$

where X' and Y' are independent copies of X and Y, respectively.

An unbiased empirical estimator of the squared MMD is

$$\widehat{\text{MMD}}^2 = \frac{1}{n(n-1)} \sum_{i \neq i'} k(X_i, X_{i'}) + \frac{1}{m(m-1)} \sum_{j \neq j'} k(Y_j, Y_{j'}) - \frac{2}{nm} \sum_{i=1}^{n} \sum_{j=1}^{m} k(X_i, Y_j).$$

Under the null hypothesis $P = Q$, the MMD equals zero when a characteristic kernel is used. Statistical significance is assessed via a permutation or bootstrap procedure, in which sample labels are randomly permuted and the test statistic is recomputed to generate an empirical null distribution and corresponding p-value.

The flexibility afforded by the choice of kernel allows the MMD test to capture a wide range of distributional differences, including higher-order moments and nonlinear dependencies. As a result, MMD has become a widely used discrepancy measure in two-sample testing, generative model evaluation, and approximate Bayesian computation frameworks.

Statistical evaluation of Crocker plot distributions for model comparison

To justify the use of Bayesian information criterion (BIC) in light of the results of Marin et al. [1], we assess whether the posterior predictive summaries generated by each model, in our case Crocker plots, are statistically distinguishable. If the samplings of Crocker plots for each model are found to be statistically distinguishable, then that means the differences between the results of the two models is great enough that the BIC method should be able to choose which model is optimal. If they are not indistinguishable, then the BIC method may not give accurate results because the results of the two models are too similar. For each row of Table 1, we considered the posterior densities (an example is shown in Fig 4) and generated 500 Crocker plots for both $\text{Model}_{\text{AL}}$and Model_{DO}. Parameter combinations were sampled according to their posterior probabilities, with acceptance probabilities proportional to the posterior density, resulting in retention of only the highest-probability parameter sets (approximately the top 1%). This procedure ensures that the resulting Crocker plots reflect the dominant posterior behavior of each model.

To compare the resulting collections of Crocker plots, we applied three complementary nonparametric two-sample tests: PERMANOVA, the Energy Distance test, and the MMD test. These tests were selected because they are well suited for high-dimensional summary statistics represented as matrices and are designed to assess differences between entire distributions rather than individual scalar values. For each test, the null hypothesis is that the distributions of Crocker plots generated by $\text{Model}_{\text{AL}}$and $\text{Model}_{\text{DO}}$are the same.

While all three methods test for distributional differences, they emphasize distinct aspects of the data. PERMANOVA assesses differences in group centroids within a specified distance space and is primarily sensitive to changes in the overall structure of the summary statistics, though it may also reflect differences in within-group dispersion. The Energy Distance test measures discrepancies between distributions based on pairwise distances and is consistent against all fixed alternatives, making it sensitive to differences in location, scale, and distributional shape. In contrast, MMD compares distributions through kernel mean embeddings and, depending on the choice of kernel, can capture higher-order and nonlinear differences that may not be detected by purely distance-based measures. Together, these tests provide a

robust assessment of distributional distinguishability across multiple complementary notions of difference.

The resulting test statistics and associated permutation-based p-values are reported in S1 Table. In all cases, the p-values are less than 0.001, providing strong evidence against the null hypothesis and indicating that the posterior predictive distributions of Crocker plots differ significantly between the two models. Importantly, these results do not quantify model quality directly but instead establish that the summary statistics used for inference are meaningfully distinct across models. This distinction is a necessary condition for principled model comparison and supports the subsequent use of BIC to score and compare models based on their posterior predictive behavior.

References

[1] Jean-Michel Marin, Natesh S. Pillai, Christian P. Robert, and Judith Rousseau. Relevant statistics for bayesian model choice. *Journal of the Royal Statistical Society Series B: Statistical Methodology*, 76:833–859, 2014.
