# Supplementary material for: Topologically-based parameter inference for agent-based model selection from spatiotemporal cellular data
Source: PLoS Comput Biol. 2026 Sep 25;22(9):e1014801. doi: 10.1371/journal.pcbi.1014801 (PMC13641708; doi:10.1371/journal.pcbi.1014801)
Supplement: Supplementary file 4 [file pcbi.1014801.s004.pdf]

S4 Appendix. Nearest-neighbor t-SNE visualizations

Supporting Information for "Topologically-based parameter inference for agent-based model selection from spatiotemporal cellular data."

Alyssa R. Wenzel, Patrick M. Haughey, Kyle C. Nguyen, John T. Nardini, Jason M. Haugh, and Kevin B. Flores

To investigate the differences between the with and without alignment groups, we modified the original t-SNE plot in Fig 2B and 2C. Instead of coloring each point by their color coordinated by its location in 3D space, we colored all points with W=0 red and all points with W=0.05 in blue (see S1 Fig). Extending it further, for each row of Table 1, we highlighted the point with the ground truth C,L,W parameter values in yellow and found its nearest neighbor in t-SNE space of the other W value.

Through this new visualization of the Crocker summaries for the two different models, we see that they occupy two distinct regions of space. This can give a user confidence that model selection is a possibility. If these two models heavily overlapped, then there might not be enough information to distinguish between the two models during the model selection process.
