# Supplementary material for: Topologically-based parameter inference for agent-based model selection from spatiotemporal cellular data
Source: PLoS Comput Biol. 2026 Sep 25;22(9):e1014801. doi: 10.1371/journal.pcbi.1014801 (PMC13641708; doi:10.1371/journal.pcbi.1014801)
Supplement: Supplementary file 5 [file pcbi.1014801.s005.pdf]

S5 Appendix. Akaike information criterion

Supporting Information for "Topologically-based parameter inference for agent-based model selection from spatiotemporal cellular data."

Alyssa R. Wenzel, Patrick M. Haughey, Kyle C. Nguyen, John T. Nardini, Jason M. Haugh, and Kevin B. Flores

Akaike information criterion (AIC) is a widely used criterion for model selection and comparison in statistical modeling and machine learning. Like BIC, it evaluates the trade-off between model complexity and goodness of fit. However, AIC is derived from an information-theoretic perspective, specifically through the minimization of Kullback–Leibler divergence, and is primarily oriented toward predictive accuracy.

The AIC is defined as:

$$\text{AIC} = -2\ln(\mathcal{L}) + 2(k+1)$$

where $\mathcal{L}$ is the likelihood of the model given the data, and k is the number of parameters in the model. The log-likelihood $\ln(\mathcal{L})$ is defined as:

$$\ln(\mathcal{L}) = -(n/2)\ln(2\pi) - (n/2)\ln(\sigma^2) - \frac{SSE}{2\sigma^2}$$

where $\sigma^2 = SSE/n$.

The penalty term $2(k+1)$ discourages overly complex models by penalizing the inclusion of additional parameters, though less strongly than the $(k+1)\ln(n)$ term used in BIC. In contrast to BIC, which is motivated by Bayesian evidence and is asymptotically consistent for model selection, AIC does not possess this consistency property and instead prioritizes predictive performance. For this reason, BIC is more aligned with the objectives of the present study. Nevertheless, AIC provides a complementary perspective, and its inclusion allows for additional comparison across model selection criteria.

The corresponding AIC model-selection results are reported in S2 Table.
