# Supplementary material for: Topologically-based parameter inference for agent-based model selection from spatiotemporal cellular data
Source: PLoS Comput Biol. 2026 Sep 25;22(9):e1014801. doi: 10.1371/journal.pcbi.1014801 (PMC13641708; doi:10.1371/journal.pcbi.1014801)
Supplement: Supplementary file 6 [file pcbi.1014801.s006.pdf]

S6 Appendix. Case analysis: When $\mathrm{Model}_{\mathrm{AL}}$ outperforms $\mathrm{Model}_{\mathrm{DO}}$

Supporting Information for "Topologically-based parameter inference for agent-based model selection from spatiotemporal cellular data."

Alyssa R. Wenzel, Patrick M. Haughey, Kyle C. Nguyen, John T. Nardini, Jason M. Haugh, and Kevin B. Flores

In Table 1, there are two cases in which $\mathrm{Model}_{\mathrm{AL}}$yields lower sum of squared errors (SSE) and BIC values than $\mathrm{Model}_{\mathrm{DO}}$, despite the data being generated using $\mathrm{Model}_{\mathrm{DO}}$. In the remaining eight cases, $\mathrm{Model}_{\mathrm{DO}}$attains lower SSE and BIC values, consistent with the ground truth model. Below, we examine the two instances in which $\mathrm{Model}_{\mathrm{AL}}$outperforms $\mathrm{Model}_{\mathrm{DO}}$under these criteria.

In the first instance, corresponding to ground truth parameters $(\mathrm{C}, \mathrm{L}) = (0.5, 0.5)$, the median parameter estimates for $\mathrm{Model}_{\mathrm{AL}}$are $(\mathrm{C}, \mathrm{L}, \mathrm{W}) = (0.751, 0.173, 0.012)$, while the median estimates for $\mathrm{Model}_{\mathrm{DO}}$are $(\mathrm{C}, \mathrm{L}) = (0.645, 0.169)$. In the second instance, corresponding to ground truth parameters $(\mathrm{C}, \mathrm{L}) = (0.2, 1.5)$, the median parameter estimates for $\mathrm{Model}_{\mathrm{AL}}$are $(\mathrm{C}, \mathrm{L}, \mathrm{W}) = (0.142, 2.02, 0.008)$, while those for $\mathrm{Model}_{\mathrm{DO}}$are $(\mathrm{C}, \mathrm{L}) = (0.202, 1.566)$. Thus, although $\mathrm{Model}_{\mathrm{AL}}$achieves lower SSE and BIC values in these two cases, its parameter estimates do not more closely recover the ground truth parameters. Rather, $\mathrm{Model}_{\mathrm{AL}}$identifies alternative parameter combinations that provide a slightly better fit to these particular simulated data sets. Notably, the estimated values of W remain close to zero, indicating that the improved fit does not require substantial alignment behavior. Because $\mathrm{Model}_{\mathrm{AL}}$approaches $\mathrm{Model}_{\mathrm{DO}}$as W approaches zero, these results illustrate that the two models can be difficult to distinguish when the inferred alignment strength is small. Thus, the SSE and BIC values remain useful measures of relative model fit, but should be interpreted together with the inferred parameter values when distinguishing between these nested models.
